# Using process drama in EFL education: A systematic literature review

**DOI:** 10.1016/j.heliyon.2024.e31936

**Published:** 2024-05-24

**Authors:** Shujie Luo, Lilliati Ismail, Norhakimah Khaiessa binti Ahmad, Qian Guo

**Affiliations:** a Faculty of Educational Studies, Universiti Putra Malaysia, Malaysia; b Faculty of Children Development and Health Management, Chongqing Vocational and Technical University of Mechatronics, China

**Keywords:** Process drama, Drama in education, EFL, Systematic literature review

## Abstract

Process drama, which emphasizes the active exploration of fictional roles and situations, has proven to be an effective pedagogical approach in language teaching and learning. Despite its recognized efficacy, the systematic evaluation of process drama's impact on English as a Foreign Language (EFL) education remains understudied. This systematic review aimed to investigate the current literature on the relationship between process drama and EFL teaching and learning. Using the keywords "process drama" and "EFL," publications released between 2003 and 2023 were meticulously extracted from various reputable databases, including ProQuest Citation, Web of Science, Scopus, Science Direct, Taylor & Francis, SAGE, and Google Scholar. In total, 30 studies (27 articles, two master's theses, and one PhD thesis) that met the inclusion criteria were analyzed comprehensively based on their primary characteristics, fostering in-depth discussions on the diverse factors influencing EFL learning and teaching through process drama. Notably, the review underscores that process drama exerts a significant and positive impact on EFL learning and teaching, particularly by enhancing language skills, students' language learning outcomes, and EFL teacher development.

## 1 Introduction

Drama has played an enduring role in education for centuries, tracing its roots back to the Middle Ages [1]. Through interactive and dynamic engagement with the audience, it serves as an authentic platform for language utilization in realistic settings [2]. As such, various categories of drama, including educational drama, creative drama, drama activities, and process drama, confer unique benefits in language education, especially in the teaching and learning of English as a Foreign Language (EFL).

In particular, process drama stands apart from conventional drama methods or scripted performances as it espouses an open-ended and creative pedagogical approach, which actively encourages learners to participate in and contribute to the co-construction of imaginative dramatic worlds [3]. As emphasized by Kramsch, language teaching transcends the mere transmission of grammatical structures and vocabulary; it also involves a nuanced process of negotiation and meaning construction [4]. In this regard, by embedding language usage within a theatrical environment, process drama affords learners the opportunity to immerse themselves in real-life communication scenarios, thereby augmenting their confidence in navigating linguistic contexts beyond the confines of the classroom. Consequently, process drama emerges as an eminently suitable pedagogical approach within EFL instruction, providing a dynamic learning environment where students can proficiently apply their language skills, engage meaningfully with their peers, and cultivate their communicative competencies.

Based on the drama teaching method, O'Neill formulated a systematic theoretical framework for process drama [5] that eschews a fixed script or predetermined outcome. This approach prioritizes the development of students' capacity to understand complex characters and situations, rather than their personal development or drama skills. Consequently, process drama diverges from traditionally isolated drama activities by constructing a representation of reality through a negotiated process led by participants [6], with an emphasis on the process rather than the final product.

Bolton encapsulated how process drama organically integrates the following characteristics: improvisation; thematic exploration; independent scene units; script generation through action; focus on participant changes; experience without written scripts; and active teacher intervention both within and outside the theater [7]. In short, instead of using isolated skits with predetermined outcomes, process drama adopts thematic exploration to discover outcomes during the process [8]. With such structure and strategies, the immersive and interactive experience of process drama holds the potential to significantly enhance the efficacy of EFL learning and teaching by elevating motivation and productivity among EFL learners in an engaging and stress-free language learning environment.

The manifold advantages of process drama have garnered recognition from numerous language researchers [[9], [10], [11], [12], [13]]. First, drama facilitates language learners' engagement in reflective, constructivist, and active learning within the classroom, which consolidates language skills by integrating verbal and nonverbal communication features [14]. Second, it enhances communication by infusing meaningful contexts into the language classroom, emphasizing the practical application of language skills [15]. Learners, through drama, can also delve into their inner thoughts, actions, and strengths, as it authentically mirrors their behaviors and personalities [16]. Moreover, drama cultivates self-confidence, self-esteem, and awareness of others, thereby fostering motivation among learners [3,17]. Consequently, it leads to learners’ heightened and more active participation in the classroom.

Overall, the incorporation of process drama in the language classroom offers diverse interactive opportunities for a broad spectrum of language functions [18]. This approach enables students to consciously use language across various contexts and accomplish a range of tasks. Therefore, process drama has progressively gained acknowledgment as a valid pedagogical approach supporting language learning in the EFL context [19,20].

### 1.1 Aim and objectives

This study seeks to conduct a systematic review of process drama studies within the EFL context over the past two decades. The primary objectives guiding this review are as follows.

(a) To explore the overall research trend of process drama within EFL education over the last twenty years.
(b) To explore the diverse influences of process drama on EFL education across different domains.

## 2 Materials and methods

### 2.1 Search keywords and databases

This systematic literature review adhered to the Reporting Standards for Systematic Evidence Syntheses (ROSES) method [21]. On May 23, 2023, a preliminary bibliographic search was conducted in the ProQuest Citation, Web of Science, Scopus, Science Direct, Taylor & Francis, SAGE, and Google Scholar databases. The search employed pertinent keywords, including "Process Drama," "Educational Drama," "Drama in Education," "Dramatic Techniques," "Drama Pedagogy," "Drama-based Pedagogy," "Drama Activities," "Drama Project," "EFL, " "ESL," "English Teaching," and "English Learning." This process yielded 189 articles, with the subsequent elimination of duplicates resulting in 131 unique search results.

### 2.2 Eligibility

In the next phase, the authors meticulously examined the manuscripts to ascertain their adherence to the predetermined inclusion criteria. Specifically, the inclusion criteria for the current review encompassed studies that met the following conditions.

a. Studies measuring EFL learning and teaching achievement through process drama;
b. Studies exploring determinants/predictors of EFL learning and teaching outcomes through process drama;
c. Studies published between January 2003 and May 2023; and
d. Studies written in English.

Conversely, studies were excluded from the present review if they.

a. Did not focus on EFL learning or teaching;
b. Reported on integrated drama techniques, where process drama constituted only a small part; or
c. Had no language learning or teaching-related outcomes.

**Fig. 1:**
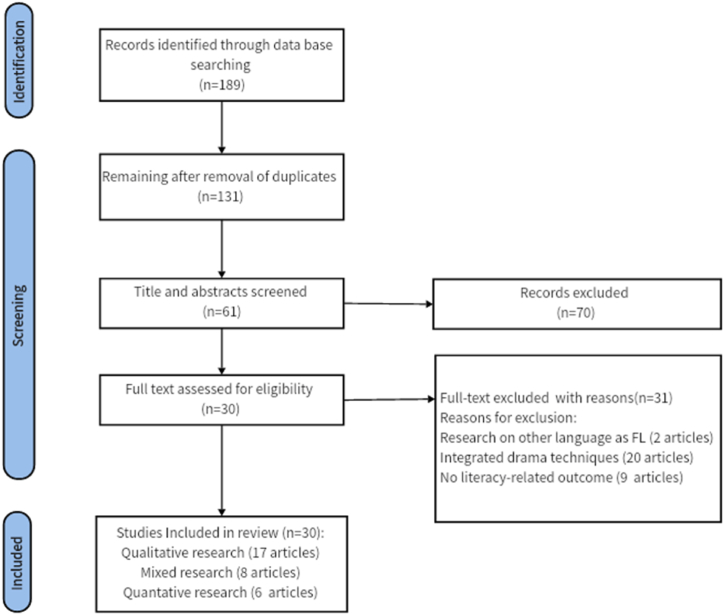
Flow diagram of the search and selection process.

A thorough screening of titles and abstracts from the initial pool of 131 articles resulted in the exclusion of 70 irrelevant articles. Subsequently, upon comprehensive content review with the application of the inclusion and exclusion criteria, an additional 31 articles were excluded. The culmination of this process yielded 30 articles eligible for the subsequent quality assessment step (see Fig. 1).

### 2.3 Data extraction and analyses

Table 1 summarizes the essential details of the included manuscripts, as follows: (a) first author; (b) publication year; (c) sample; (d) setting; (e) research method; (f) determinants/predictors measures; and (g) major outcomes. Accuracy checks were rigorously conducted by two independent reviewers who assessed all phases of the systematic review. The selected publications also underwent thematic examination in accordance with the guidelines of Kiger and Varpio [22].

**Table 1:** Summary of reviewed studies.

First Author	Sample	Place of Study	Research Method	Variance	Outcome
Publication Year					
Stinson (2006) [23]	N = 140 High school students	Singapore	Mixed	Oral communication	PD project has a positive effect on students' English oral communication skills.
Cameron (2007) [24]	N = 27 Primary students	Canada	Qualitative	Reading comprehension	Drama enables students with disabilities to improve their reading comprehension, and allows opportunities for dialogues.
Dora To (2011) [25]	N = 160 Primary teachers	Hong Kong, China	Qualitative	Teacher development	Teachers perceive PD as a method of providing authentic learning experiences.
Kao (2011) [26]	N = 30 College students	Taiwan, China	Mixed	Questioning techniques	EFL drama activities facilitate more interactive questioning than traditional approaches.
Ntelioglou (2011) [27]	N = 50 Adult students	Canada	Qualitative	Multimodal language learning	Drama pedagogy provides situated practice and multimodal representations of meaning.
Anderson (2012) [19]	N = 16 Primary students	USA	Mixed	Written language	PD transfers students' contextualized writing to decontextualized genre writing.
Dunn (2012) [28]	N = 15 refugee children	Australia	Qualitative	Digital technologies Language learning	Technology serves seven key functions within PD.
Chen (2013) [29]	N = 27 high school students	Taiwan, China	Qualitative	Critical intercultural awareness	PD allows language learners to develop flexibility and mobility in critical perspectives.
Gill (2013) [30]	N = 10 Undergraduates	Australia	Quantitative	Oral English	Drama has a positive effect on international students' English oral skills.
Donnery (2014) [31]	N = 32 Undergraduates	Japan	Mixed	Communicative competence	PD has a positive influence on Japanese university-level EFL learners' linguistic and intercultural competence.
Atas (2015) [32]	N = 24 High school students	Turkey	Mixed	Speaking anxiety	Drama techniques significantly lower the speaking anxiety of EFL learners.
Park, (2015) [33]	N=NA Undergraduates	Korea	Qualitative	Benefits of drama projects	Drama projects are an effective means of positive attitude change and of promoting cognition, positive affect, and social skills.

First Author	Sample	Place of Study	Research Method	Variance	Outcome
Publication Year					
Stinson (2015) [34]	N = 22 Primary students	Australia	Qualitative	Oral communication	PD contributes positively to students' oral communication skills and intercultural awareness.
Çoban (2017) [35]	N = 27 Undergraduates	Turkey	Mixed	Communication strategies	Participating in PD does not have a statistically significant effect on EFL learners' use of communication strategies.
Ding (2017) [36]	N = 21 Postgraduates	China	Qualitative	English speaking	PD has a positive effect on classroom relationships, emotional arousal, and students' English speaking skills.
Galante (2017) [37]	N = 24 Undergraduates	Brazil	Quantitative	L2 Oral	Drama-based instruction can lead to significantly larger gains in L2 English oral fluency.
Yılmaz (2017) [38]	N = 23 9th grade students	Turkey	Qualitative	Students' attitude English learning	B1 and A2 Turkish EFL students have gained a positive attitude towards English learning through drama.
Araki (2018) [39]	N = 30 Undergraduates	Japan	Qualitative	Speaking anxiety and confidence	PD can increase students' speaking motivation and assist their conceptual understanding of global issues.
Gabitova (2018) [40]	N = 60 Undergraduates	Russia	Quantitative	Communication skills	Drama techniques promote the development of quick thinking, creativity, and emotional expressiveness.
Galante (2018) [12]	N = 24 EFL learners	Brazil	Mixed	L2 speaking anxiety	Drama can enhance comfort levels when speaking L2.
HiŞmanoglu (2019) [41]	N = 42 K-12 teachers	Turkey	Quantitative	English speaking	K-12 EFL teachers are highly aware of the impact of using drama to develop students' speaking skills.
Hulse (2019) [42]	N = 42 Student teachers	England	Qualitative	Creativity Innovation	Teachers' capacity to use PD is dependent on their personal experiences and dispositions as well as the support of their school.
Alam (2020) [43]	N = 1003 Undergraduates	India	Quantitative	Instructional methods Process drama techniques	Role-play and enactment lead to speaking progress. Unscripted activities develop cooperative learning.
Araki (2020) [44]	N = 40 Primary school teachers	Japan	Qualitative	Language anxiety	Teachers have an unrealistic expectation of their English capabilities. PD helps teachers reconnect pedagogical proficiency and agency.
Murray (2021) [45]	N = 6 University teachers	Japan	Qualitative	Process Drama Practitioners English teaching	Prior positive experiences with drama encourage teachers' adoption and self-directed initial use of PD in teaching practices.

First Author	Sample	Place of Study	Research Method	Variance	Outcome
Publication Year					
Alam (2022) [9]	N = 1003 Undergraduates	India	Quantitative	Accuracy & fluency Non-verbal skills	Drama activities develop students' communicative skills, critical thinking, and non-verbal language skills.
Ding (2022) [11]	N = 18 primary school teachers	China	Qualitative	Teacher development	Teacher needs effectively link language and drama, but there are three main difficulties.
Uştuk (2022) [46]	N = 20 Junior pre-service teachers	Turkey	Qualitative	Language teaching identity	Drama can provide learning experiences for pre-service teachers to navigate language teacher identity tensions.
Yang (2022) [47]	N = 2 classes Undergraduates	China	Qualitative	Reading strategies and skills	The integration of drama activities into EFL reading class can motivate students to read and actively participate in reading class.
Wells (2023) [13]	N = 35 undergraduates and postgraduates	New Zealand	Mixed	Students' perception of PD Embodied learning	ITE students' experiences of PD on campus constitute a powerful form of embodied learning.

Note: PD is Process Drama.

## 3 Results

The results of this review first focused on the primary characteristics of the included articles, namely their publication year, academic setting, and analytical methods. The pivotal findings from the reviewed studies were then thoroughly discussed in the context of the factors influencing EFL learning and teaching through process drama. Subsequently, coding and thematic analysis revealed three principal themes that illustrate the efficacy of process drama in EFL learning and teaching: language skills, students' affective factors, and teacher development.

### 3.1 Overall research trends

**Fig. 2:**
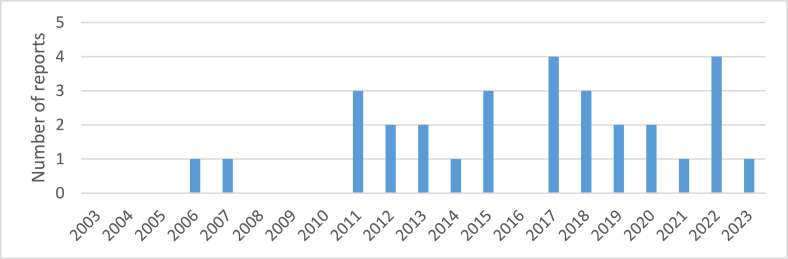
Number of studies from 2003 to 2023.

Table 1 presents an overview of the population, location, research method, variables, and outcome of the 30 reviewed studies. In terms of number of studies over the past 20 years, the search results show a relatively stable publication rate, with no particular peak or pattern in publication period (see Fig. 2).

**Fig. 3:**
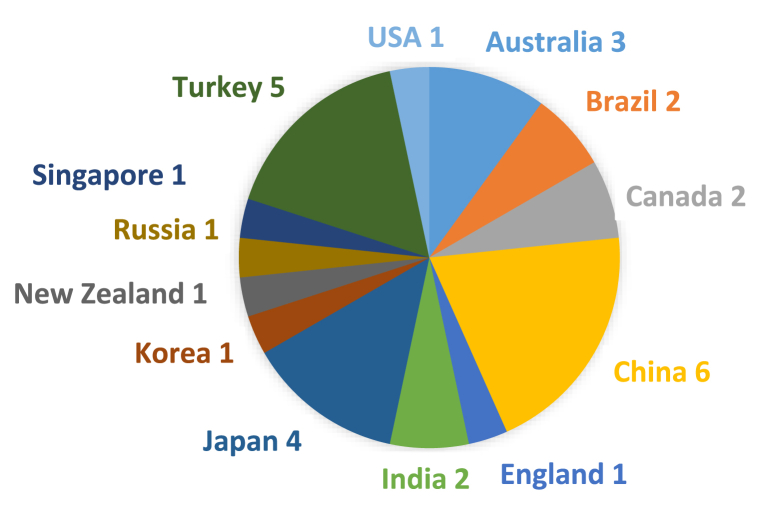
Research location of selected studies.

As Fig. 3 demonstrates, resent research on the application of process drama in the EFL context has been conducted across 13 different countries, namely Singapore, Canada, China, USA, Australia, Japan, Turkey, Korea, Brazil, Russia, England, India, and New Zealand. Among these, China (Mainland China = 3, Hong Kong = 1, Taiwan = 2), and Turkey (n = 5) have produced the most publications, followed by Japan (n = 4).

**Fig. 4:**
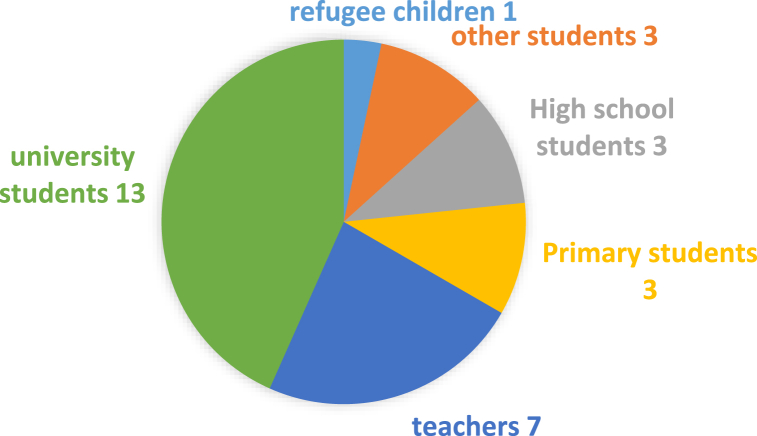
Samples of selected studies.

Next, Fig. 4 indicates that the majority of the studies were conducted among university students, with 10 studies on undergraduates, one on college students, one on postgraduates, and one on both undergraduates and postgraduates. A significant portion of research has also sampled teachers from different levels, including four studies on primary teachers, two on pre-service teachers, and one on university teachers.

The results further show that scholars have employed various methods to explore and analyze the effects of process drama on EFL. Notably, it appears that the existing literature has predominantly adopted a qualitative lens to present the varied perspectives of teachers and students or to describe process drama implementation. In fact, 53 percent of the reviewed studies utilized qualitative methods like interviews, classroom observations, focus groups discussions, and reflective journals. On the other hand, eight studies employed a mixed-method design and six used a quantitative methodology. Only a few, however, have provided experimental evidence to elucidate the effects of process drama on English learning. While Atas [32], Anderson [19], Gill [30], and Kao, Carkin and Hsu [26] did apply experimental or quasi-experimental designs, they had no control groups for comparison and did not provide substantive evidence to ascertain whether drama-based pedagogy is especially efficacious for English learning.

### 3.2 Influences of process drama on EFL education

#### 3.2.1 Process drama and language skills

Twelve of the 30 reviewed studies scrutinized the impact of process drama on students' language skills across various educational levels (e.g., primary, high school, undergraduate, and postgraduate). These investigations lend credence to James Moffett's assertion [48] that process drama serves as the cornerstone for all language activities, encompassing speech, writing, and reading. Nonetheless, the majority of the 12 studies predominantly concentrated on the effects of process drama on students' speaking skills [9,11,23,30,31,34,37,40,41], while two evaluated students' reading skills [24,47] and only one examined students' writing skills [19].

In Anderson's research [19], process drama was implemented among 16 primary students in the USA over eight weeks. The findings indicated that process drama facilitates the transfer of students' contextualized writing to decontextualized genre writing. Notably, contextualized in-role writing experiences exert a mediating effect on students' written linguistic specificity and productivity. Another study by Cameron applied process drama among primary students for one academic year to enhance reading comprehension [24]. The results demonstrated that process drama not only improves reading comprehension but also provides opportunities for dialogues, connecting students to the text in meaningful ways. Similarly, in Yang's study of Chinese EFL undergraduates, integrating process drama into the EFL reading class emerged as a motivating factor for students to read actively and participate more in reading sessions, cultivating their enhanced understanding of the text and improved reading strategies during the process [47].

As process drama emerges as a potent tool for engaging students in language learning, numerous studies have explored its impact on communicative skills, specifically speaking and listening [23,30,31,34,40,40,41]. The integration of drama into foreign language teaching fosters a stress-free and enjoyable environment, making it easier for students to speak the target language [49]. According to Dundar, process drama offers students extensive opportunities for communication and interaction [50], enabling them to overcome their shyness and focus on the language; in turn, this reinforces their engagement in communicational activities.

In the EFL classroom, speaking serves as the verbal conduit for students to communicate with one another; however, many struggle with dialogue and expressing their thoughts [51]. Process drama addresses this issue by immersing learners in both as-if and real-life situations, equipping them with a communicative and meaningful approach to speaking [18]. Hulse and Owens proposed that learners with minimal foreign language proficiency can actively participate in open-ended process drama with adequate linguistic support [3], facilitating their communication through a blend of verbal and non-verbal responses, even within a restricted linguistic range.

Given its evident advantages, researchers are increasingly focusing on the effectiveness of process drama in the domain of speaking [28,34,36,39,43]. Coleman's quasi-experiment on Korean EFL students demonstrated significant pretest-posttest improvements in English speaking skills following a five-day intensive drama-based program [52]. Stinson's explanatory case study of an Australian primary school proposed the use of process drama in a dialogic classroom, identifying four dimensions of oral communicative skills: functional, dialogical, linguistic, and paralinguistic. Her findings revealed a positive contribution of process drama to students' oral communication skills and intercultural awareness [34]. Similarly, Stinson and Freebody's study on EFL students in four Singapore high schools reported notable pretest-posttest gains in English speaking skills with process drama classes [23], positing process drama as a robust teaching strategy for oral language instruction across various settings. However, it is worth noting that while they introduced a "oracy" checklist, there was no formal pre- and post-test to assess participants' oral communication skills, suggesting a need for further analysis and development.

#### 3.2.2 Process drama and cognitive factors

Among the 30 reviewed articles, five suggest that process drama has the potential to decrease the affective filter of EFL learners, enabling them to shed inhibitions and overcome shyness and anxiety [12,32,33,38,39]. By establishing specific classroom conventions, positive and cooperative behaviors become more easily cultivated. This teaching process immerses students in an enjoyable environment where involvement is essential. If implemented effectively, it can aid students in developing positive attitudes toward learning a foreign language [53].

In Park's process drama project, a positive correlation was observed between process drama and EFL learning attitude [33]. Conducting three case studies in Korea, she found that process drama can be successfully used in diverse teaching situations, serving as an effective means of fostering positive attitude change. Yılmaz and Dollar's study with 23 ninth-grade students in Turkey further supported this notion, revealing that students of different language proficiency levels can develop a positive attitude towards English learning through drama [38].

As process drama provides a comfortable communication environment for EFL learners, it has also been employed to reduce students' speaking anxiety levels [12]. Araki and Raphael investigated the effect of process drama on EFL learners' English-speaking anxiety in a Japanese international university [39]. Their results indicated that process drama can be applied as a pedagogy to increase students' motivation for speaking and enhance their conceptual understanding of global issues. A similar finding was noted in Atas' study in Turkey [32]. However, limited research provides a clear explanation of how process drama influences various cognitive factors (e.g., motivation, attitude, and anxiety) simultaneously in English speaking learning, as well as how these effects interplay throughout the EFL learning process.

#### 3.2.3 Process drama and teacher development

Six studies included in this review investigated the effects of process drama on teacher development [3,11,25,[44], [45], [46]]. Araki revealed that Japanese teachers often harbor unrealistic expectations regarding their English capabilities [44]. However, engagement in process drama shifts their mindset, helping them reconnect to pedagogical proficiency and agency. Additionally, Dora To implemented a year-long process drama program with 160 English teachers in Hong Kong, revealing numerous benefits, including increased motivation to learn, improved confidence in speaking, heightened engagement among students of varying abilities, and enhanced teacher-student relationships [25]. Nevertheless, Hulse and Owens pointed out in their study that an individual teacher's capacity to use process drama is dependent on personal experiences, dispositions, and the extent of support received in school [3]. Ding also highlighted the need for teachers to adopt a changed perspective and acquire knowledge of drama pedagogy to effectively link language with drama [11]. She summarized three main difficulties in this teaching process: text selection and interpretation, translation into drama activities, and classroom implementation.

Uştuk demonstrated the potential of process drama to highlight the juxtaposition between EFL teacher identity and tensions [46]. His findings revealed that pre-service EFL teachers perceive tensions as valuable learning opportunities in dramatized settings. Exploring these tensions in a safe learning environment through make-believe activities allows participants to navigate through them and (re)construct their teacher/learner identities through identity work in metaxis. Furthermore, Murray, Reis-Jorge, and Regan's study examined language teachers' experiences of process drama in Japan [45]. Their in-depth interviews with six process drama practitioners indicated that positive student outcomes and feedback are crucial for teachers to continue using process drama as a teaching practice.

#### 3.2.4 Process drama and other educational benefits

Other studies have related process drama to improvements in more specific areas of language education, such as questioning techniques [26], multimodal language learning [27], critical intercultural awareness [29], and communication strategies [35]. Dunn, Bundy, and Woodrow even applied process drama through digital technologies, finding it to be an excellent opportunity for refugee children to gain agency over their own learning and create shared experiences with classmates and teachers [28]. Alam, Karim, and Ahmad further pointed out that through the application of process drama among students, role-play and enactment lead to speaking progress, unscripted activities develop cooperative learning, and non-verbal activities improve body language [43]. Lastly, Wells, Sandretto, and Tilson revealed that students are able to merge theory and practice through embodied learning in process drama practice [13].

## 4 Conclusion and suggestions for future research

With the aim of exploring the implications of process drama in the EFL learning and teaching domain, this systematic review has synthesized 30 relevant studies from the last two decades. The body of work indicates that the core strengths of process drama lie primarily in enhancing EFL learners' language skills, affective language learning factors, and teacher development. As a process-oriented drama pedagogy, process drama provides a rich context for numerous language encounters and fosters authentic discourse between teachers and students [34].

In the application of PD within EFL contexts, several challenges persist. First, teachers must adopt a transformed perspective and acquire knowledge on drama pedagogy, recognizing its interdisciplinary nature and the need for seamless integration of language and dramatic techniques. Unlike traditional drama performance, the selection and interpretation of text, translation into dramatic activities, and classroom implementation should be adapted to process-oriented and student-centered features [11]. Second, successful PD implementation requires students ' comprehension and immersion in the dramatic milieu, allowing them to fully express their subjectivity and actively engage in the learning process. Given the constraints of limited teaching time, such as a single lesson, achieving optimal outcomes becomes improbable. Therefore, for future applications of PD, the provision of additional resources by relevant experts and researchers to support practical educators is imperative for the effective enactment of PD methodologies.

Furthermore, most studies suggest that process drama is an effective teaching approach in EFL classrooms, particularly by creating a positive and communicative learning environment for speaking. However, no investigation into the impact of process drama on English speaking skills has been conducted in mainland Chinese universities, especially among students with relatively low English speaking competence and high language learning anxiety. Students’ affective factors also need to be taken into account in this domain, as they could have different effects on students' learning processes and outcomes. Therefore, to gain greater insights into the nature of process drama, future research should concurrently analyze the interplay between students' affective factors (e.g., motivation, attitude, and anxiety) throughout the learning process. In addition, there is a need for more comparative empirical evidence on how students' English learning outcomes differ between traditional teaching approaches and the process drama approach. Finally, researchers could integrate quantitative and qualitative data in their analyses to provide a more comprehensive understanding of how process drama supports EFL learners' language learning mechanisms.

## Data availability

The data pertaining to this study have not been deposited in a publicly accessible repository, given that all relevant data are thoroughly detailed in the article, supplementary materials, or appropriately cited in the manuscript.

## CRediT authorship contribution statement

**Shujie Luo:** Writing – review & editing, Writing – original draft, Validation, Resources, Project administration, Methodology, Formal analysis, Data curation, Conceptualization. **Lilliati Ismail:** Supervision, Conceptualization. **Norhakimah Khaiessa binti Ahmad:** Supervision. **Qian Guo:** Writing – review & editing, Formal analysis.

## Declaration of competing interest

The authors declare that they have no known competing financial interests or personal relationships that could have appeared to influence the work reported in this paper.

## References

[1] Stern S.L. (1983).

[2] Lee Y.J., Liu Y.-T. (2022). Promoting oral presentation skills through drama-based tasks with an authentic audience: a longitudinal study, asia-pacific edu. Res..

[3] Hulse B., Owens A. (2017). Process drama as a tool for teaching modern languages: supporting the development of creativity and innovation in early professional practice. Innovat. Lang. Learn. Teach..

[4] Kramsch C. (2002). Language and culture. https://books.google.com.my/books/about/Language_and_Culture.html?id=XRPiONIC2PMC&redir_esc=y.

[5] O'Neill C. (1995). http://archive.org/details/dramaworldsframe0000onei.

[6] Taylor P., Warner C.D. (2006). Structure and Spontaneity: the Process Drama of Cecily O'Neill, Trentham.

[7] Bolton G. (1979). https://archive.org/details/towardstheoryofd0000bolt.

[8] Piazzoli E. (2012).

[9] Alam S., Al-Hawamdeh B.O.S. (2022).

[10] Araki N. (2021). Drama pedagogy as a catalyst for shifting language anxiety in primary school teachers: offering critical engagement within EFL classroom. Qual. Res. J..

[11] Ding L. (2022). The form and meaning: when English language teachers learn to teach through drama: language, Culture, Literature. Scenario.

[12] Galante A. (2018). Drama for L2 speaking and language anxiety: evidence from Brazilian EFL learners. RELC J..

[13] Wells T., Sandretto S., Tilson J. (2023). Bridging the theory-practice divide in teacher education through process drama pedagogy: “You fully experience what you’re learning,”. Teach. Teach. Educ..

[14] Maley A., Duff A. (2005).

[15] Wongsa M., Son J.-B. (2020). Enhancing Thai secondary school students' English speaking skills, attitudes and motivation with drama-based activities and Facebook. Innovat. Lang. Learn. Teach..

[16] Zaghloul H.S. (2018). Using creative educational drama to enhance self-development skills for the students at university level. Ijacsa.

[17] Kayaoğlu M.N., Sağlamel H. (2013). Students' perceptions of language anxiety in speaking classes. Journal of History Culture and Art Research.

[18] Kao S.-M., O'Neill C. (1998).

[19] Anderson A. (2012). The influence of process drama on elementary students' written language. Urban Educ..

[20] Alam S. (2022). Imagine, integrate, and incorporate: English language and its pedagogical implications in EFL classrooms. Rupkatha J. Interdiscip. Stud. Humanit..

[21] Haddaway N.R., Macura B., Whaley P., Pullin A.S. (2018). ROSES RepOrting standards for Systematic Evidence Syntheses: pro forma, flow-diagram and descriptive summary of the plan and conduct of environmental systematic reviews and systematic maps. Environ. Evid..

[22] Kiger M.E., Varpio L. (2020). Thematic analysis of qualitative data: AMEE Guide No. 131. Med. Teach..

[23] Stinson M., Freebody K. (2006). The dol project: the contributions of process drama to improved results in English oral communication. Youth Theat. J..

[24] Cameron K.J. (2007).

[25] Dora To L., Phoebe Chan Y., Lam Y.K., Tsang S.Y. (2011). Reflections on a primary school teacher professional development programme on learning English through Process Drama. Res. Drama Educ.: The Journal of Applied Theatre and Performance.

[26] Kao S.-M., Carkin G., Hsu L.-F. (2011). Questioning techniques for promoting language learning with students of limited L2 oral proficiency in a drama-oriented language classroom. Res. Drama Educ.: The Journal of Applied Theatre and Performance.

[27] Ntelioglou B.Y. (2011). ‘But why do I have to take this class?’ The mandatory drama-ESL class and multiliteracies pedagogy. Res. Drama Educ.: The Journal of Applied Theatre and Performance.

[28] Dunn J., Bundy P., Woodrow N. (2012). Combining drama pedagogy with digital technologies to support the language learning needs of newly arrived refugee children: a classroom case study. Res. Drama Educ.: The Journal of Applied Theatre and Performance.

[29] Chen I.W.-L. (2013).

[30] Gill C. (2013). Enhancing the English-language oral skills of international students through drama. ELT.

[31] Donnery E. (2014). Process drama in the Japanese university EFL classroom: the emigration project: language, culture, literature. Scenario.

[32] Atas M., Isman A. (2015). Ministry of National Education - Turkey.

[33] Park H. (2015). Student perceptions of the benefits of drama projects in university EFL: three case studies in Korea. Engl. Teach. Pract. Critiq..

[34] Stinson M. (2015). Speaking up about oracy: the contribution of drama pedagogy to enhanced oral communication. Engl. Teach. Pract. Critiq..

[35] Çoban P. (2017). The effect of process drama on English as a Foreign Language (EFL) learners’ use of communication strategies, Doctoral Dissertation. Bilkent Universitesi (Turkey).

[36] Ding L. (2017). A reflective case study on effective English speaking through process drama. The Journal of Drama and Theatre Education in Asia..

[37] Galante A., Thomson R.I. (2017). The effectiveness of drama as an instructional approach for the development of second language oral fluency, comprehensibility, and accentedness. Tesol Q..

[38] Yılmaz G., Dollar Y.K. (2017). Attitudes of Turkish EFL learners towards the use of drama activities in English. Hasan Âli Yücel Eğitim Fakültesi Derg..

[39] Araki N., Raphael J., Ruegg R., Williams C. (2018). Teaching English for Academic Purposes (EAP) in Japan.

[40] Gabitova L., Shayakhmetova L., Beisembayeva Zh (2018). The effectiveness of drama methods in the development of communication skills. Revista Publicando.

[41] HiŞmanoğlu M., Ҫolak R. (2019). A study on Turkish EFL teachers' perspectives on using drama to develop students' speaking skills in the EFL classroom. Novitas-ROYAL (Res. Youth Lang.).

[42] Hulse B., Owens A. (2019). Process drama as a tool for teaching modern languages: supporting the development of creativity and innovation in early professional practice. Innovat. Lang. Learn. Teach..

[43] Alam S., Karim M.R., Ahmad F. (2020). Process drama as a method of pedagogy in ESL classrooms: articulating the inarticulate. JECS.

[44] Araki N. (2020). Drama pedagogy as a catalyst for shifting language anxiety in primary school teachers: offering critical engagement within EFL classroom. Qual. Res. J..

[45] Murray K., Reis-Jorge J., Regan J.-A. (2021). Becoming a process drama practitioner: an exploratory study of higher education language teachers in Japan. Lang. Teach. Res..

[46] Uştuk Ö. (2022). Drama-in-teacher-education: a ‘metaxical’ approach for juxtaposing EFL teacher identity and tensions. Lang. Teach. Res..

[47] Yang X. (2022). Constructivism-based drama activities in EFL reading classes. TESOL J..

[48] Moffett J. (1973).

[49] Göktürk Ö., Çalışkan M., Öztürk M.S. (2020). The effects of creative drama activities on developing English speaking skills. Journal of Inquiry Based Activities.

[50] Dundar S. (2013). Nine drama activities for Foreign Language classrooms: benefits and challenges. Procedia - Social and Behavioral Sciences.

[51] Bsharat T.R.K., Barahmeh M.Y. (2020).

[52] Coleman L.E. (2005).

[53] Batdı V., Batdı H. (2015). Effect of creative drama on academic achievement: a meta-analytic and thematic analysis. Educ. Sci. Theor. Pract..

